# Identification of Design Requirements for a Software Application for Use by Clinicians That Collects Acute Stroke Treatment Data During Clinical Workflow: Pilot Study

**DOI:** 10.2196/64800

**Published:** 2025-12-19

**Authors:** Adam Forward, Gizem Koca, Aymane Sahli, Noreen Kamal

**Affiliations:** 1 Department of Industrial Engineering Faculty of Engineering Dalhousie University Halifax, NS Canada

**Keywords:** data collection, medical technology, simulated field test, software, user interface design

## Abstract

**Background:**

Clinical registries are critical for monitoring processes of care in diseases and driving quality improvements. However, many smaller hospitals lack the required resources to collect the necessary data to contribute to registries.

**Objective:**

This study aims to design and evaluate a data collection tool for acute stroke treatment that streamlines the collection of process data and provides tools to aid clinician users while not interfering with clinical workflow. The evaluation will identify key design requirements that facilitate prospective data collection and add value for clinicians.

**Methods:**

We developed a prototype tool for testing using Figma Pro for use on an iPad. Clinicians were recruited through convenience sampling to test the prototype’s use in a small-scale simulated clinical field experiment, during which participant were asked to think aloud and then complete a series of tasks to mimic a mock stroke treatment while inputting the required data into the prototype. Follow-up semistructured interviews were conducted to gain feedback on how the prototype integrated into the workflow and on the aspects of the prototype they felt helped and hindered their use of it. Qualitative data analysis combined review of the experiment recordings to identify the most frequent errors made during the scenario and deductive thematic analysis from the follow-up interviews to determine user needs for the following prototype iteration. The insights from the feedback identified design requirements that were implemented in the iterated design and documented to provide a reference for future product designers.

**Results:**

Three participants were recruited from 2 hospitals between April 18 and June 6, 2024, for the simulated field experiment. The scenario took 10-12 minutes, with 1.2-3.7 minutes spent using the prototype, depending on whether optional features such as the NIHSS (National Institute of Health Stroke Scale) calculator were used. The simple and condensed layout and features such as NIHSS calculators, benchmark metric timers, and the final pop-up summary received the most positive feedback from each participant. Issues identified included small target sizes causing higher error rates, lack of color in important features reducing their visibility, and grouping of mandatory and optional information field layouts leading to a disjointed flow. The key design requirements include prioritizing simple dynamic layouts, sufficient target sizes to prevent errors, useful features with clear visual cues, and prompt data feedback to facilitate seamless integration.

**Conclusions:**

A prospective data collection tool for clinicians to use during stroke treatment can add value for clinicians and, with further testing, can be integrated into workflow. The design requirements identified through this study can provide a basis for streamlining the collection of accurate data while increasing the value of the tool for users and should be considered by future product designers to add value to their software and improve user experience.

## Introduction

Clinical registries are databases that hospitals use to monitor disease and delivery of care to better understand outcomes and drive improvements. They aggregate data containing patient demographics, medical history, hospital processes, and outcomes of care to measure the quality of care and guide improvements. The use of clinical registries has been shown to improve the quality of care, especially in chronic diseases such as cancer and diabetes [1], but they are also beneficial in acute diseases such as stroke [2].

Multiple stroke registries have demonstrated success in improving treatment processes. One of the largest clinical registries used in stroke treatment is Get With the Guidelines–Stroke in the United States, which combines registry data collection and feedback with hospital toolkits, stakeholder meetings, and collaborative workshops to guide improvements in enrolled hospitals and has significantly increased the rates of patients receiving treatment while ensuring that adverse events remain low [3]. Other large-scale stroke registries that have been associated with improved quality of care include the Registry of Stroke Care Quality in Europe [4,5] and the Australian Stroke Clinical Registry [6]. Finally, in Canada, the current national clinical registry for stroke is OPTIMISE (Optimising Patient Treatment in Major Ischemic Stroke With Endovascular Thrombectomy) [7,8], which records the care path of patients that are treated with endovascular thrombectomy.

For a stroke registry to successfully highlight the processes of care, it must have accurate data so stakeholders can make informed decisions on how to improve their processes. Data collection for stroke is typically conducted retrospectively through chart reviews; however, prospective data collection for stroke has been practiced in various centers [9]. Both retrospective and prospective data collection methods are resource intensive and require staff dedicated to either abstracting or collecting the data, respectively. Because of the resource-intensive nature of data gathering and the time sensitivity necessary to treat a stroke, it is difficult for smaller hospitals with fewer clinicians to contribute to clinical registries.

In Canada, the stroke centers currently contributing data to OPTIMISE are comprehensive stroke centers (CSCs) [7,8]. These centers are frequently larger academic teaching hospitals with the resources available to collect stroke treatment data. However, primary stroke centers (PSCs), which are capable of treating patients with thrombolysis only, are typically small community and rural hospitals. These hospitals make up most stroke treatment centers in Canada; yet, most are unable to contribute data to OPTIMISE under the current system data collection for stroke registries. Many PSCs do not have the resources required to retrospectively abstract stroke treatment data. Additionally, prospective data collection at a small hospital could risk taking time from patient care during the treatment process to input data without dedicated staff.

Novel methods to streamline data collection for stroke treatment using software applications has been studied [10,11], and while there has been success in reducing treatment times, multiple issues have arisen related to data completion and low clinician user satisfaction due to the added work to existing processes [11,12]. The ability to collect accurate and complete data while minimizing the added workload and improving user satisfaction is crucial for prospective registries to succeed in sustainably driving quality improvement initiatives.

There are known barriers to the adoption of data collection technologies in health care settings, including cost, technical training, time, usefulness, and personnel availability [13,14]. By reducing these barriers, adoption of novel clinical technology can increase through improvements in user experience and accessibility for data collection. The use of software to aid in prospectively collecting data during acute stroke treatment can facilitate contributing data to clinical registries. However, there is currently an unmet need to understand the design of a tool that can aid in the prospective collection of data during the acute stroke treatment process.

There is a knowledge gap in understanding the design requirements of a software interface, such as the information layout, interaction styles for inputting information, desired features, and feedback mechanisms that facilitate seamless prospective data collection for clinician users in the acute stroke treatment process and minimize barriers to clinician use. While existing mobile software has been tested and barriers to technology adoption have been studied, there has been no analysis of the specific features and barriers that affect clinician users’ ability and satisfaction in integrating a prospective data collection software into the stroke treatment workflow.

This study proposes a prototype data collection tool that is designed, validated, and evaluated by clinician users to specify the key design requirements for collecting data during the workflow of the acute stroke treatment process. The aim of the prototype is to achieve 2 main goals: first, to allow seamless and accurate collection of prospective stroke treatment data, and second, to provide features within the prototype that offer value to clinician users. If these 2 conditions are met, it is anticipated that clinicians will be more open to adopting the technology and integrating it into their stroke treatment operations.

## Methods

### Overview

This research is an exploratory study anchored in the design thinking process. The process includes 5 stages: empathize, define, ideate, prototype, and test. The empathize and define stages of design thinking were completed in a previous study through the completion of semistructured interviews [15]. This study focuses on the ideation, prototyping, and testing of the data collection prototype.

The first iteration of the prototype used a combination of established usability rules, existing literature on health care software, results from previously conducted semistructured interviews [15] analyzing differences in the processes of stroke centers across Canada, and expert opinions from members of the Canadian Stroke Consortium, a group of stroke professionals who agreed on the variables required for the stroke registry. The prototype was designed to incorporate data capture for all key data metrics agreed on by the Canadian Stroke Consortium, and it was then tested by clinician users who provided feedback to identify key design requirements to improve the tool’s usability and utility for a second iteration. These requirements were then integrated into the prototype for future testing.

### Ideation

Key considerations when ideating this prototype included the information to be collected in the software, required features, and the layout and organization of information. The prototype was first developed as low-fidelity pen-and-paper designs and then upgraded to a medium-fidelity prototype using Figma.

#### Preidentified Requirements

The information for the prototype corresponds with established performance benchmarks in stroke treatment [16,17]. The required information was determined by members of the Canadian Stroke Consortium. Two variable lists were created based on the stroke center classification (PSCs and CSCs) and were used to design the information architecture and determine the structure of the prototype. The complete variable lists can be found in Multimedia Appendix 1.

The previous study identified features that clinicians stated would facilitate use of the prototype and aid in seamless integration with workflow. Five main aids were discussed as adding value for clinicians in stroke treatment: a calculator to determine the National Institute of Health Stroke Scale (NIHSS), inclusion-exclusion criteria to determine a patient’s eligibility for treatment, a summary of the information collected, a “now” button to collect process times, and 2 on-screen timers recording the time since patient arrival and benchmark times.

#### Information Architecture

An information architecture (IA) was developed to define the layout and organization of the prototype and is provided in Multimedia Appendix 2 (Figure S1). The main wireframe contains the information fields on a single page; thus, as opposed to the standard use for navigation, this IA highlights the content and organization of the main page. The IA was organized into 3 sections: patient and stroke information, hospital process, and treatment process. Two sub-IA’s were included to show the structure of the NIHSS calculator and the inclusion-exclusion criteria for treatment. The sub-IA’s (Figures S2 and S3 in Multimedia Appendix 2) highlight the order of the information given in the featured tools and the terminology for each information field.

### Prototyping

The foundation of the design was anchored in Nielsen’s [18] usability heuristics. A stroke treatment data collection software naturally requires speed and learnability, so some heuristics were prioritized in the initial design. For example, 3 of Nielsen’s heuristics prioritized in the design included “match between the system and the real world,” “flexibility and efficiency of use,” and “aesthetic and minimalist design.” These heuristics were most critical in minimizing the user’s cognitive load and ensuring they would not be overwhelmed while inputting data into the software.

#### Pen and Paper Designs

The low-fidelity prototype was created using the pen-and-paper software called Concepts (TopHatch Inc). The design considered the data fields, features, required number of screens, and navigation. The pen-and-paper designs were developed by AF and reviewed by NK.

#### Medium Fidelity Prototype

The final pen-and-paper design was upgraded to a medium-fidelity prototype using Figma Pro (Figma Inc; 2016). The medium-fidelity prototype was built for use on an iPad to test how the interactivities and modality integrated into the clinical workflow for users. The prototype contained working links and conditional variables that updated in real time to act as a properly functioning application.

### Testing

#### Recruitment

The research study was conducted at Dalhousie University in Nova Scotia, Canada. The geographic location limited the clinician sample pool, as the province has a small population with only 1 CSC and 2 stroke-trained neurologists. Additionally, the research team had to ensure that each participant completed the experiment under the same conditions and with the same equipment, so participation was required to be in person. A participation invitation was sent to any prospective participant who had a direct role in the acute stroke treatment process and was within driving distance of the research laboratory where the study took place. Two hospitals—a CSC and a thrombolytic-capable center (which was often bypassed due to its proximity to the CSC)—were within a reasonable driving distance of the research laboratory. There were no additional screenings for eligibility, as the sampling pool was already limited by geographic location, participant expertise, and the required time to participate. Because of these factors, convenience sampling was used to recruit clinician participants. Participants were invited by email to participate in the user testing session.

#### Simulated Scenario

The user testing environment was prepared to ensure that the prototype was evaluated in a scenario that best mimicked the stroke treatment process with respect to the space and equipment available while minimizing any human risk. The simulated environment was prepared to ensure that participants used the prototype while moving around, carrying objects, and speaking to people but did not replicate other aspects of an emergency department, such as background noise or navigating corridors. A single room was set up with arrows to guide the participant to each area. The room was divided into areas related to hospital departments involved in acute stroke treatment. The scenario required a set of preprepared materials, including a mock patient chart, a prearrival form, and a mock thrombolytic syringe. Two volunteers participated as actors and took different roles throughout the scenario, including the roles of paramedic, emergency nurse, computed tomography (CT) technologist, and interventional radiologist. The actors were provided one of two unique scripts, depending on whether the participant was from a PSC or a CSC, to communicate information to the participant in a manner similar to how they would during a real stroke. Participants were required to complete all mandatory data fields during the scenario.

The simulated scenario was recorded using a Meeting Owl camera (Owl Labs; 2014), allowing for a 360° view of the room. The scenario and follow-up interviews were audio- and video-recorded. Automatic transcription software was used to record the participants’ feedback. Participants were asked to “think aloud” as they completed the simulated scenario, with reminders given throughout as necessary.

#### Qualitative Feedback

Follow-up one-on-one interviews were conducted with the participants to obtain their feedback on the prototype. An interview guide was used to ensure consistency in topics discussed with participants, and to minimize bias by ensuring each question was asked impartially. The interview guide is provided in Multimedia Appendix 3. The questions concerned understanding the participants’ thoughts on the layout, features, viability of integrating the prototype into workflow, and possible improvements as they completed the scenario. The feedback from both the simulated scenario and follow-up interviews was transcribed on the researcher’s laptop, manually reviewed for accuracy, and then coded using NVivo (QSR International Pty Ltd).

#### Data Analysis of Recordings

Two recordings were analyzed to evaluate the prototype: a room recording of the simulated scenario and a screen recording of the iPad. They were reviewed concurrently to evaluate the interactions made, time spent using the prototype, errors made, and total time completing the simulated scenario. The time spent using the prototype was determined by timestamping each required task’s start and end times, noting the participant’s first and last interactions with the prototype for each task, and then adding all task times. The number of errors made was identified through a combination of the screen recording, using a Figma feature that highlights the screen when a misinput is made, and manual review of the simulated clinical scenario.

Deductive thematic analysis was conducted for the interviews, referencing Nielsen’s heuristics for the codebook. Each participant transcript was analyzed line by line, with codes assigned to participant quotes representing an idea from one of Nielsen’s heuristics. Each code was labeled as a “positive” or “negative” application of Nielsen’s heuristics, where positive uses were to be kept for following prototype iterations and negative uses were to be changed for the following iteration. Participant quotes that best captured the group consensus on each aspect of the prototype were selected, then grouped by the primary investigator and discussed with the research team to determine the underlying heuristic the design features promoted. Because this was a pilot study and the themes were predetermined, no interrater reliability was conducted. Additionally, this study was not intended to be an exhaustive study reaching thematic saturation. However, the primary investigator ensured that feedback reported in the Results was discussed by at least 2 participants to identify overlap in participant feedback. The features and design requirements were analyzed and categorized to determine the considerations, features, and design choices for future iterations.

### Ethical Considerations

Ethical approval was obtained from the Dalhousie University Research Ethics Board for prospective research involving human subjects. The study protocol, with REB file number 2023-6753, was approved by the Dalhousie Ethics Board. All participants provided recorded verbal informed consent before participating in the study, in accordance with the requirements of the Dalhousie Ethics Board. Participants were informed that participation was voluntary and that they could withdraw from the study at any time; that recordings of the experiment would be taken for analysis; that follow-up interviews would be transcribed and deidentified by coding to protect their privacy; and that confidentiality would be preserved when reporting results. Participants were also informed that no compensation would be provided for study participation.

## Results

### First Iteration Design

The first iteration was designed to be simple, intuitive, and efficient. Five types of input elements were included for data entry: manual input (ie, typing), buttons, steppers (ie, buttons that increase or decrease an initial value by one using a “+” or “-” button), time pickers (ie, a slider that uses a finger swipe to change an initial time value), and drop-down menus. These input elements were chosen based on 3 main factors: anticipated time to enter the information, the amount of screen space used, and the variable data types (integers, alphanumeric, list, etc). Default values were agreed upon for 3 fields to reduce error potential and interaction time: all date fields would default to “today’s” date; arrival method would default to “Ambulance,” as that is the primary method of arrival for stroke patients; and the “CTA/CTP occurred immediately after CT” would default to “yes.”

The presentation of information was defined in this stage. To reduce the cognitive load for users, expandable information fields were designed based on user inputs. This included the “treatment information” section not expanding until the user chose the treatment method, and if CT angiography (CTA) or CT perfusion (CTP) did not occur immediately after the CT scan, the form would expand to require inputting the CTA or CTP time.

**Figure 1:**
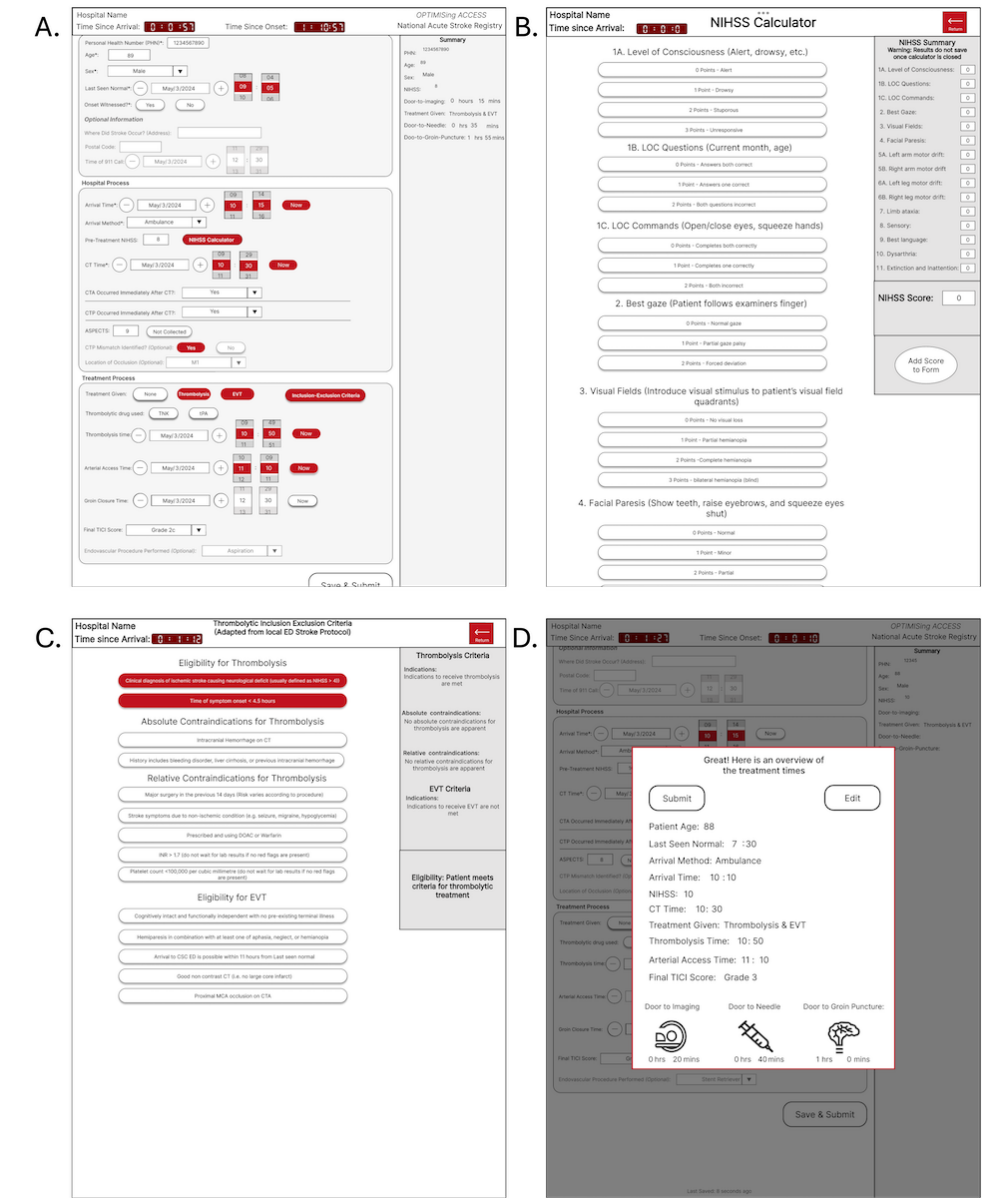
First prototype iteration screenshots, including: (A) main data entry page with sample information filled, (B) National Institute of Health Stroke Scale (NIHSS) calculator feature, (C) Inclusion-exclusion criteria feature, and (D) final summary pop-up.

A final summary was included to provide feedback to the user after the data were collected. Two methods to summarize the information were used: a sidebar summary that condensed the information of the main page for easier readability and a final pop-up summary at the end providing the main information documented and benchmark times. Both methods were included to determine participants’ preferences during user testing. Figure 1 shows the layout and features of the first iteration.

### User Testing

#### Simulated Scenario

Recruitment occurred between April 18, 2024, and June 6, 2024. During that time, 3 clinicians were recruited to complete the simulated clinical scenario from 2 different hospitals—one CSC and one PSC. The participants consisted of an emergency physician, a stroke-trained nurse, and a neurologist. Each simulated scenario completed by the participants took 10-12 minutes. The total time using the prototype ranged from 1 minute and 12 seconds to 3 minutes and 43 seconds.

The longest task time was observed for participants who chose to use the NIHSS calculator, which was completed by 2 of the 3 participants (taking 1 minute and 50 seconds and 2 minutes and 8 seconds of the prototype use time). The cumulative number of tasks completed by the participants for each input element and comparisons in the time taken to complete tasks with each related input element are provided in Table 1. A 2-sample *t* test (2-tailed) was completed comparing related interaction styles (ie, data fields that could be filled in multiple ways, such as by manual typing or selecting a premade list). No interaction style had a statistically significant difference in task times, except for the comparison of buttons to drop-down menus (*P*=.008).

**Table 1:** Comparison in the cumulative number of tasks per input element completed by the participants and comparisons of the mean, SD, 95% CI and *P* value comparing for each related interaction style.

Comparison group and interaction style			Frequency, n		Task time (seconds), mean (SD)		95% CI		*P* value
**Group 1**									.25
	Manual input	12		4.17 (2.91)		2.31-6.02			
	Time picker	16		5.50 (2.92)		3.92-7.08			
**Group 2**									.008
	Button	11		1.46 (0.66)		1.63-1.86			
	Drop-down menu	8		4.50 (2.27)		2.60-6.40			
**Group 3**									.30
	Time picker	16		5.50 (2.92)		3.92-7.08			
	Drop-down menu	8		4.50 (2.27)		2.60-6.37			
**Group 4**									.60
	Button	11		1.46 (0.66)		1.63-1.86			
	Stepper	6		1.67 (0.82)		0.81-2.52			

The number of errors made by each participant was reviewed and categorized. Six different types of errors were identified. “Missed target” errors occurred when a participant attempted to select or interact with a data entry field but missed the target box. Missed target errors for each interaction style (manual input, time picking, dropdown menu, and buttons) were recorded separately to determine which interaction styles were most prone to errors. When combined, missed targets made up 80% (32/40) of the identified errors. Typing errors were exclusive to manual input fields and occurred when participants activated the manual input correctly but entered incorrect information into the main form. Finally, “feature missed” errors occurred when participants did not recognize a feature they had to use, such as the “save and submit” button, or did not use a feature during the field study that they stated in the follow-up interviews they would have used if they recognized it. An average of 13 errors were made per participant. Table 2 provides the number of errors of each type made by each participant.

**Table 2:** Number of errors made by each participant (P1-P3) by error type, categorized by interaction style: manual information fields (typing), dropdown menus, time pickers (sliders), and buttons. Missed targets are counted individually.

Error type	Error frequency, n			
	P1	P2	P3	Total
Missed target (info field)	3	7	6	16
Missed target (dropdown)	1	3	5	9
Feature missed	1	3	2	6
Missed target (time)	3	1	0	4
Missed target (button)	2	1	0	3
Typing error	1	1	0	2

#### Qualitative Feedback

Follow-up interviews conducted with participants took between 60 and 75 minutes. The interviews reviewed each aspect of the prototype with participants to gather their feedback. Participants’ responses were coded using Nielsen’s heuristics as a framework to evaluate how they felt each aspect of the prototype met the heuristic criteria.

The most consistently positive responses about the prototype among all 3 participants regarded the minimalist design, which presented information only as needed; the usefulness of the provided features, including the NIHSS calculator, inclusion-exclusion criteria, and “now” buttons; and the value of the final pop-up summary. Participants felt that the organization of information made inputting the information intuitive and simple.

I think that it captures really good information. It is very clear, it is a one-pager, succinct, I like the collapsible boxes, it is very organized. It is neat and it is in an order that makes sense.

Participant 3

The timers, NIHSS calculator, and inclusion-exclusion criteria features included in the design were well received by all 3 participants. Tools such as the NIHSS calculator were already being used in participants’ workflows, and they discussed using their phones to check the time throughout the process, so having the main tools available in one place was beneficial to participants.

The timers, the NIHSS calculator, the inclusion exclusion criteria, those are the big things that I liked.

Participant 2

When discussing the potential integration of the prototype into clinical workflow, the main facilitating factor identified was the pop-up summary. Two participants noted that if the final summary could be emailed to colleagues, printed, or linked to a patient’s chart, they would be more likely to use the prototype.

That is a beautiful summary to take a quick screenshot of and be like, “hey, I have everything I need for that patient in terms of their ASP (acute stroke protocol). So, I love the summary.

Participant 1

The most negatively received aspects of the prototype discussed by all 3 participants were the small target sizes causing errors, small text sizes reducing readability, and the lack of visual cues in the design. The error potential in the design was the most frequently recognized issue discussed by participants, as it led to longer use times and increased frustration.

I think just everything is pretty small and you have to be very accurate as to where you push, and then if you are just a bit off, it won’t accept that you are doing something.

Participant 3

A similar issue discussed concerned the readability of the text. Two participants found that if they wanted to review their information, they would need to focus on the iPad screen rather than the next task in the scenario. This concern was also applicable to the timer feature, as it was not immediately apparent how much time had passed when clinicians glanced at the iPad, reducing the usefulness of the feedback the software attempted to provide. Additionally, for the timers, participants stated that an added indication of how much time had passed would be useful.

I would make [the text] a bit bigger, a bit easier to see on the go because I find I was really concentrating on trying to read, as opposed to walking.

Participant 1

I would make the timers bigger. The time since arrival, time since onset counters, and maybe even some indication of how close we are to the benchmarks.

Participant 2

Another issue regarding the lack of recognizable cues concerned the navigational features in the prototype being easy to miss. Two participants used the NIHSS calculator feature, but both hesitated to press the “add score to form” button due to its lack of color and its placement on the side of the screen. Additionally, the inclusion-exclusion criteria feature was missed by one participant because its location in the “treatment process” section did not align with their mental workflow, in which they began determining eligibility for treatment as soon as the patient arrived.

(When referring to the NIHSS calculator) It is probably a bit more intuitive to have a button on the bottom, and then maybe a different color so that it is a bit more obvious.

Participant 3

[I did the] Inclusion and exclusion criteria, in my head, I think of it earlier on in the medical history taking, so I would have that closer to the top.

Participant 2

Two other issues with the layout discussed were the placement of optional information and the efficiency of the drop-down menus. All 3 participants found the first set of optional information fields interfered with the flow of how information was presented. They discussed that, while the sections made sense, the last seen normal information naturally felt as though it should be followed by the arrival time.

To me, intuitively, the information with last seen normal, was the onset witnessed, I gather once the patient is in hospital. So, I think of those pieces together. So, the fact that they are divided is not the most intuitive way that I think about it.

Participant 3

The other concern with the layout involved the drop-down menus. Two participants found that the dropdown menus were unnecessary for data fields that had only a few options. They felt that buttons displaying all options or checkboxes would be easier to use and more efficient.

CTA and CTP would be easier if it was just a checkbox, like did CTA occur? Yes, instead of needing to select a drop-down, it would just be a little bit faster.

Participant 2

All 3 participants discussed their concerns with integrating the first prototype iteration into their workflow. Four points were discussed as requirements for integrating the software into a hospital: the ability to reliably save data, return to previously incomplete records if retrospective data collection is needed, add comments for outlier records, and reaccess the data. Participants discussed the inclusion of an autosave feature to ensure that collected data would not be lost if an extenuating circumstance arose.

I think one of the hopes would be that this would have some interval save function so that if say, you pass it off to somebody else, and they just set it down, and the iPad in the interval has died, that the information does not immediately disappear.

Participant 3

The ability to return to incomplete records was discussed as an important element. Although all 3 participants agreed that most information could be gathered in real time, concerns arose that if certain data elements—either those captured by existing electronic software (such as CT time) or those with difficult-to-identify starting points (such as groin puncture time)—were to be captured in real time, it might lead to discrepancies in the data.

Even if you are standing in the room, the interventionalist is not really announcing when they punctured [the groin] and when they have closed, and so that would have to come from the interventionalist sheet. And, although the ‘now’ buttons are good, I think that it would be difficult for those to be recorded in real time.

Participant 3

Two participants discussed the expansion of the prototype into workflow and how its value could be improved if a free-text section were added to comment on cases. Participants explained that patient factors can influence the time to treat a patient and that a way to comment on how these factors influenced the treatment times could be beneficial when the data are reviewed.

A quick comment to say, ‘the transfer got delayed by a snow blizzard’ or ‘treatment was a little weird’, a little comment box to just say those things that are not captured here that come up would help.

Participant 1

The final topic discussed regarding integration of the software was the ability to reaccess the data. Participants found the idea of real-time feedback of the process through the pop-up summary beneficial, but they also wanted the ability to return to the data even after the information was completed.

A question I would have is where can I get this data back? That would be a very important question, because the biggest complaint I have is we put all this work in to get data and then we are not allowed to access it.

Participant 1

### Second Iteration Design

**Figure 2:**
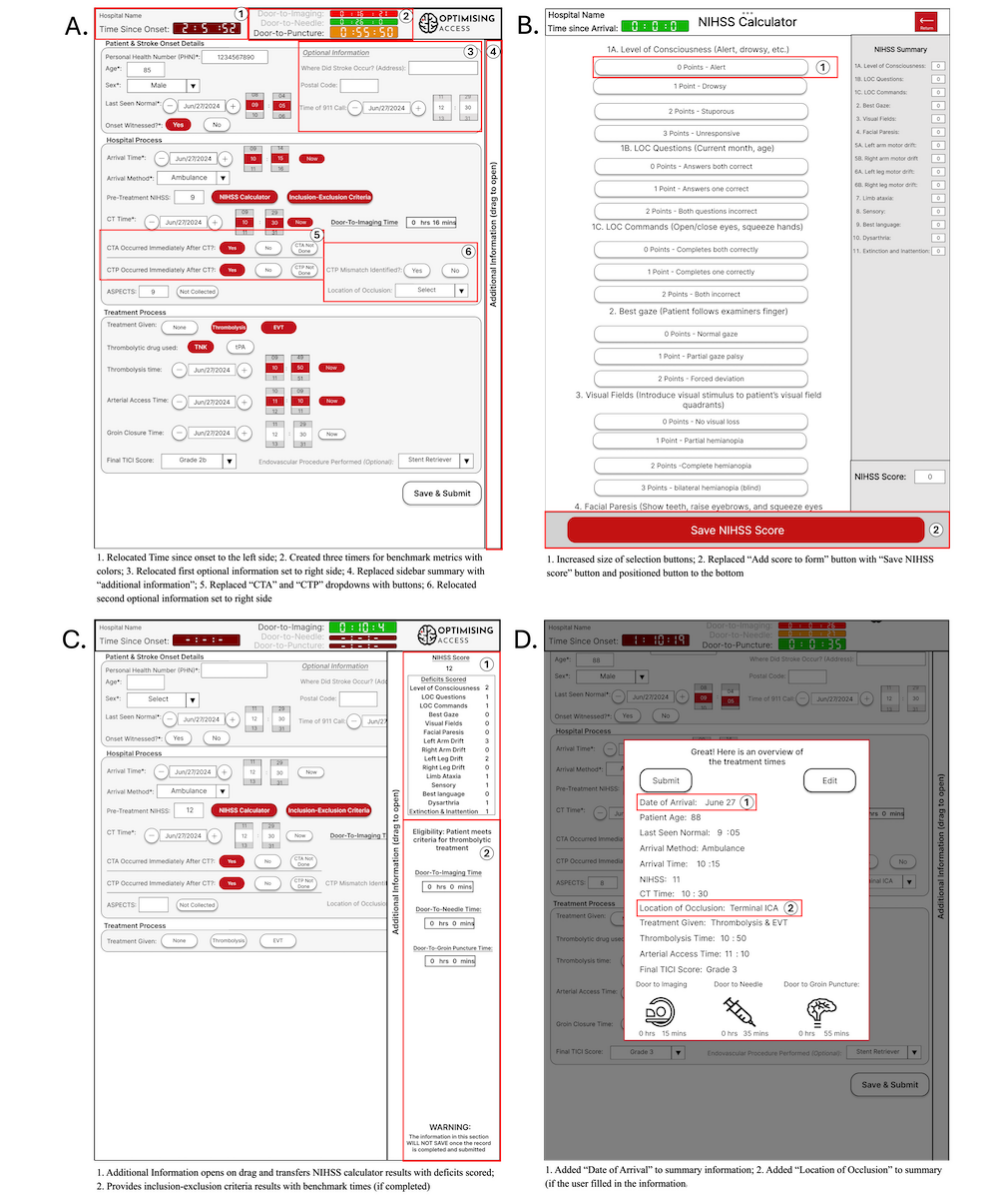
Second prototype iteration screenshots including: (A) main page with relocated information, timers with colors, and new buttons replacing dropdown menus; (B) National Institute of Health Stroke Scale (NIHSS) calculator with larger selection buttons and a “Save NIHSS” button located at the bottom; (C) new “additional information” slider displaying the NIHSS scores and inclusion exclusion criteria; and (D) modified final summary including the date of arrival and location of occlusion.

A second iteration of the prototype was designed accounting for the main usability issues identified in the simulated field experiment. The revised design, including the layout and modified features, are shown in Figure 2. The main changes for the second iteration of the prototype aimed to reduce the error potential for selecting data fields and improve the visibility of the system status with visual cues. About 80% of the errors made occurred due to missing an interaction target, indicating the need to increase the size of the targets. However, the information presented still had to be condensed to a single page, and there was no available space to increase the target sizes, since the sidebar summary used approximately 25% of the screen space. Therefore, the sidebar summary was removed to provide more screen space for the main page. The additional space was used to reorganize the layout of the main page by moving the optional information fields to the right-hand side. This allowed the heights of the manual input fields to be increased from 30 pixels (approximately 0.8 cm) to 45 pixels (approximately 1.2 cm) to reduce potential error rate and increase user satisfaction, while keeping the content of the prototype condensed to one page.

To improve visual cues, color, size and location were considered the primary additions to increase the visibility of important features. The “inclusion-exclusion criteria” button was moved to the “hospital process” section beside the “NIHSS calculator” button to correspond with the users’ mental model. In the NIHSS calculator, the “add score to form” button was replaced with a “Save NIHSS score” button at the bottom of the screen and colored red to further increase visibility. For the timers, 3 colors were used to visually signify to the user how close they were to meeting the treatment benchmark times, and 2 timers were added to correspond with the 3 benchmark metrics. Green signified the user is well within the benchmark, orange indicates the user was nearing the benchmark, and red indicated the user had missed the benchmark.

Finally, the dropdown menus for the “CTA/CTP occurred immediately after imaging” fields were replaced with buttons. The remaining dropdown menus were not replaced either due to the lack of screen space or the number of options within the dropdown menu.

### Design Requirements

The results of the user testing identified key requirements for the acute stroke treatment data collection software to effectively integrate into clinical workflow. Textbox 1 provides a summary of the design requirements gathered from the user tests and follow-up interviews, as well as their categorization using Nielsen’s heuristics as a framework, highlighting how they add value to the design.

**Design requirements identified and categorized by Nielsen’s heuristics.**

**Visibility of system status**

- Final pop-up summary of information entered, and times collected throughout the process that can be saved and shared
- Timers counting arrival and onset times with visual cues corresponding to treatment benchmark times

**Match system to the real world**

- NIHSS (National Institute of Health Stroke Scale) calculator and inclusion-exclusion criteria features available in “Hospital process” section
- Separate mandatory and optional data fields to create a logical information sequence

**User control and freedom**

- Provide a way to search and review previously completed records
- A free-text comment system to note unique cases

**Consistency and standards**

- Position navigation buttons at the top or bottom of the screen
- Provide an autosave feature (and a manual save option)

**Error prevention**

- Target sizes must be greater than 50 pixels in width and 30 pixels in height
- Add color to navigation buttons for recognizability
- Provide default values based on date, expected method of arrival, and hospitals’ scanning capabilities

**Recognition rather than recall**

- Transfer results of NIHSS calculator with deficits scored and inclusion-exclusion criteria features to main page

**Flexibility and efficiency of use**

- Font sizes greater than 14 point for quick scanning
- Use buttons in place of dropdown menus (where space allows)
- “Now” buttons to timestamp processes (with time pickers to allow adjustability if the time was missed)

**Aesthetic and minimalist design**

- Keep information condensed to one page
- Have information fields expand and collapse based on user inputs

**Help users recognize, diagnose, and recover from errors**

- Allow an incomplete record to be saved, highlighting the missing information fields

## Discussion

### Principal Findings

This qualitative study identified and categorized design requirements for a prototype software aimed at collecting data during clinical workflow through an exploratory small-scale user test. The results highlight some of the unique needs of clinicians recruited to collect stroke treatment data prospectively in a software application. Usability testing guidelines recommend 5 users to identify 80% of usability issues and conducting multiple small-scale tests to achieve the best iterative results [19]. Although only 3 users were able to test the first prototype iteration due to the limited participant pool for in-person participation with a naturally busy clinician demographic, the results from the simulated scenario and follow-up interviews provided rich data in a more accurate context in which the final software is anticipated to be used compared with a stationary usability test, and overlapping insights concerning the usability of the prototype were identified. Additionally, by setting up the experiment environment so that participants tested the prototype while moving and talking to multiple people while inputting data, the feedback and requirements gathered reflected the needs of clinicians in a scenario more accurate to the real-world context in which the software is ultimately intended to be used compared with a remotely conducted usability test. In this discussion, we compare the requirements gathered from the participants and features implemented in the prototype in relation to previously identified techniques to improve data quality and stroke treatment processes. Additionally, we discuss potential ideas to iterate the final version of the software to incorporate design requirements that could realistically scale for real-world adoption.

### Facilitators for Improved Data Quality

The value of the prototype depended on the ability to efficiently capture information. An obvious consideration was the speed of data entry by minimizing time spent using the prototype. The design aimed to balance a condensed, simple layout with all pertinent information on one page of the software; however, this increased the likelihood of errors due to small target sizes that must be tapped with the user’s finger. Other unique insights that were critical when considering the usefulness of the software in the context of stroke data collection included the importance of clear visual cues, adaptable design, immediate feedback, and the clear information grouping. Previous studies have emphasized the importance of adaptability in data collection tools, allowing users to bypass information fields that are not relevant to them [20].

A feature discussed that was not implemented in the second prototype iteration was the inclusion of free-text comments. While viewed as potentially beneficial for data analysis, some concerns arise due to the time required to input free-text comments during workflow and the impact this could have on data quality. A previous study identified excessive free-text entry as a contributor to poor data quality in a stroke registry [21]. Additionally, much of the information found in the patient chart is not necessary for registry data and hinders the standardized format. If the final version of the software were to be used by multiple centers, it is important to ensure that the information captured is standardized, as it could potentially be shared to compare metrics between different hospitals. However, the inclusion of free-text fields could be combined with built-in iPad features such as a microphone to allow hands-free notetaking, and free-text comments could be a private field viewable only by specific clinicians for security purposes if deemed valuable and necessary to integrate with clinical documentation.

### Usability Issues

Despite aspects of the prototype being positively received, there were important issues concerning the prototype’s usability that impacted the participants’ perspective on integrating the first iteration into workflow. Primary issues included the size of interaction targets affecting task times, use of screen space for unnecessary features, lack of visual cues for aspects such as navigation and system feedback, unintuitive sequence of information groups causing hesitance during use, and less efficient interaction styles that required excessive inputs and increased task times. Several of the identified issues could be considered by future designers of mobile medical software beyond stroke treatment, who should consider the context in which clinicians will need to input data to identify specific needs for these usability aspects.

The biggest usability issue was the size of interaction targets. The small size of targets for manual information fields, time picking, and drop-down menus caused participants to require more time to complete tasks by reselecting targets and increased frustration during use, which reduced participant buy-in. Additionally, if implemented on a larger scale, a higher error potential could reduce overall data quality. The small target sizes were largely due to the aim of fitting the prototype’s content to a single page; however, the “sidebar summary” was removed, as participants did not view it as adding value due to its redundancy in presenting easily accessible on-screen information. After removing the sidebar summary, the information was reorganized to allow enough space to increase the target sizes while still containing the content on a single page.

Another usability issue that particularly affects the usefulness of a prototype for prospective stroke data collection is the visual cues. Due to the time sensitivity of stroke treatment, it is critical that a clinician does not have to thoroughly inspect software to understand the information they need to input, how to access a feature they want to use, or where to find information they want to review. The main issue identified with the prototype’s first iteration was that the visual cues were not clear enough to immediately signify important information to users. This included not knowing how to navigate or activate features due to lack of color in buttons, not knowing how close they were to meeting different stroke treatment benchmarks using the timers, and being unsure if a data field was mandatory or optional.

A consideration for the final software (and future testing) is training to understand how to interpret the visual cues, as they are meant to indicate how close someone is to meeting benchmark treatment times, but not as a measure of clinician performance. While the cues could help promote a sense of urgency, they are not intended to make the user feel as if they have failed when the timer turns red. Therefore, it is important to train clinician users to understand that the visual cues are purely a signifier of time and not an indirect judgment of their performance.

Finally, the information grouping and interaction styles contributed to making the first iteration less efficient. The information was presented linearly, with all data fields positioned on the left-hand side, but the order of information and lack of distinction between mandatory and optional data fields interrupted the participants’ thought process, as they assumed the next data field would be the next mandatory data point in the treatment process. Additionally, the drop-down menus (which also had higher error rates) were less efficient to use than buttons. While drop-down menus are useful in some cases, converting them to buttons allows for faster interactions and minimizes use time without compromising data quality. Design guidelines do not specify exact criteria for using drop-down menus compared with buttons; however, drop-down menus are often considered clunky if not used to conserve screen space [22].

### Features Integrated Into Stroke Treatment Processes

The NIHSS calculator, inclusion-exclusion criteria, benchmark timers, and pop-up summary at the end of the scenario were seen as value-added features rather than distractions. The use of these tools has been shown to improve treatment times [15,23], indicating that additional tools may be added in future prototype iterations if they provide value to the users. However, to accommodate the variety of potential hospitals and clinician experience levels (with both technology and stroke treatment), it was important to keep features optional to allow for the most universal appeal. Additionally, any future added features must be tested and verified with clinicians, as the concise and condensed layout was considered a critical design requirement by participants. For the PSC version of the software, a potential value-added aid could include a method of rapid telestroke consultation, as effective use and minimum delay time in telestroke consultations have been associated with improved patient outcomes [24-28]. This could be achieved by having the final software link contacts of on-call physicians to use FaceTime or perform a phone assessment, streamlining the process for completing telestroke assessments in rural and remote centers.

This study aimed to understand whether it is realistic for clinicians to prospectively collect stroke data during clinical workflow without interfering with the treatment process. While there were reservations concerning the potential to distract users, the opportunities associated with integrating the software into practice were not ignored. The pop-up summary was seen as an effective incentive for using the software during clinical workflow if it could be shared with the stroke team to provide feedback and saved to hospital systems to potentially reduce documentation load. Extensive research has shown that prompt data feedback is an effective strategy for improving treatment times [21,23,29-31]. Furthermore, a previous study indicated that documentation is one of the most time-consuming tasks for nursing staff [32], so the ability to either link the pop-up summary to a hospitals electronic medical record (EMR) or to print the summary as a sticker to attach to the patient chart would be a significant facilitator for adopting data collection software [13,33]. The final version of the software would ideally connect with the hospitals EMR, but an alternative option could be to program the final software to link with Apple AirPrint, allowing the iPad to connect with a hospital printer. This method would be easily accessible and beneficial for hospitals that have not yet converted to EMRs.

### Limitations and Future Directions

This study has several limitations. First, the prototype was designed only for the iPad, so there was no phone version to compare modality preferences. In a future test, two versions of the prototype could be designed for use on an iPad and an iPhone, both incorporating the design requirements (within their limitations), to determine how each modality impacts user experience.

The second limitation was that the sample size of 3 participants means that the findings may not be generalizable, and the results should be interpreted with caution. Additionally, it meant there was not a sufficiently large amount of task time data to find statistically significant differences between most interaction styles. With a larger sample size, more requirements are expected to be gathered, validated, and compared, so the results of this study should be taken as exploratory results that can provide direction for future software considerations but should not be seen as definitive. Further testing with additional clinicians of different demographics, experience, and geographic location can determine additional findings.

A third limitation was that the convenience sampling method can lead to selection bias, as the interested participants may not be indicative of the greater clinical population. Convenience sampling was chosen because the natural pool of potential participants was quite small, as they had to be clinicians within the study’s small, low-population province who were directly involved in stroke treatment, within driving distance of the research laboratory, and available to participate during the one-and-a-half-month timeframe allotted for user testing. However, this method was chosen to complete the simulated clinical field test, which allowed for recordings of both the prototype screen and the participants’ movement for more accurate analysis of when participants would use the prototype during the simulated scenario. Additionally, all requirements identified had to be independently identified by at least 2 (but more often all 3) participants to be considered. Future tests could recruit more participants by having a longer timeframe for recruitment, having the research team travel directly to hospitals for testing sessions, or providing incentives to recruit a larger sample for usability tests. Additionally, a larger sample can be gathered through online surveys with clinicians to review the future iteration’s tools and features.

The fourth and final limitation is that the simulated clinical scenario was conducted in a single room with individual participants with no background noise. This environment is not similar to a real emergency department, and a more realistic simulated scenario with added stimuli could result in identifying additional design requirements. To further develop the prototype, a larger in-person field test with multiple hospitals in different regions, taking place at the participants’ hospital of practice, will help identify further design requirements in the user’s environment. A high-fidelity prototype should be created using the data gathered, and the following prototype can then be tested in a real emergency department, either as a more advanced pilot test or as a clinical trial.

### Conclusions

In this study, we conducted a small-scale exploratory test to identify key design requirements for prospective data collection software to integrate into the clinical workflow of the acute stroke treatment process. User testing in a simulated clinical scenario was conducted to provide participants with a more accurate sense of how the prototype would integrate into workflow, so that feedback would be more accurately reflect the needs of clinicians in a stroke treatment scenario. Deductive thematic analysis highlighted the main factors of the prototype that both positively and negatively affected clinicians’ ability to capture data and their overall experience using the prototype. The first prototype iteration met clinician participants’ requirements of providing prompt data feedback and a simple layout. However, issues concerning high error potential, lack of visual cues to highlight system status, and information grouping hindered the ability to collect required information seamlessly, reduced the value of some features, and negatively affected the user experience. The user testing and analysis resulted in a list of design requirements and how they improve the prototype’s usability, which was then implemented in a second prototype iteration. This study identified preliminary key needs for adopting prospective data collection software and the requirements to improve the software’s value to users without hindering their workflow. The requirements identified and heuristics most applicable to the software’s features could serve as a basis for future developers when designing similar software.

## Appendix

Multimedia Appendix 1 List of variables.

Multimedia Appendix 2 Information architecture.

Multimedia Appendix 3 Interview guide.

## Abbreviations

CSC: comprehensive stroke center
CT: computed tomography
CTA: CT angiography
CTP: CT perfusion
EMR: electronic medical record
IA: information architecture
NIHSS: National Institute of Health Stroke Scale
OPTIMISE: Optimising Patient Treatment in Major Ischemic Stroke With Endovascular Thrombectomy
PSC: primary stroke center
