# Supplementary material for: Identification of Design Requirements for a Software Application for Use by Clinicians That Collects Acute Stroke Treatment Data During Clinical Workflow: Pilot Study
Source: JMIR Form Res. 2025 Dec 19;9:e64800. doi: 10.2196/64800 (PMC12759296; doi:10.2196/64800)
Supplement: Multimedia Appendix 1 [file formative_v9i1e64800_app1.pdf]

OPTIMISING ACCESS

# List of Variables

## PRIMARY STROKE CENTRE

### PATIENT INFORMATION
- **Patient Identifier**
- **Age** (Numeric)
- **Sex** (Male/Female/Other)
- **Gender** (Woman/Man/Non-binary/Other)
- **Race** (List)

### STROKE OCCURENCE INFORMATION
- **Postal Code**
- **Where did stroke occur?** (Address)
- **Last Seen Normal** (Date/Time) – – **Witnessed Onset?** (Yes/No)
- **Stroke Occurred in?** (Community/ Hospital)
- **Pre-treatment NIHSS Collected?** (Yes/No) → *IF YES* → **Pre-treatment NIHSS** (0-42 Integer)

### PRE-TREATMENT PROCESS
- **Time 911 Call** (Date/Time)
- **Arrival** (Date/Time)
- **Arrival Method** (Ambulance/Walk-in/Transfer)
- **CT** (Date/Time)
- **Was a CTA done immediately after CT?** (Yes/No/Not done) → *IF NO* → **CTA** (Date/Time)
- **Was a CTP done immediately after CT?** (Yes/No/Not done) → *IF NO* → **CTP** (Date/Time)
- **ASPECTS Collected?** (Yes/No) → *IF YES* → **ASPECTS** (0-10 Integer)
- **Location of Occlusion** (Pick list)

### TREATMENT PROCESS
- **Thrombolysis Received?** (Yes/No) → *IF YES* → **Thrombolysis** (Date/Time); **Thrombolysis Drug?** (tPA/TNK)
- **Treatment with telemedicine?** (Yes/No)
- **Patient Transferred for EVT?** (Yes/No) → *IF YES* → **Departed PSC** (Date/time); **Transfer Modality?** (Ground/Rotary/Fixed wing)

### ACUTE CARE
- **NIHSS at 24 hours collected?** (Yes/No) → *IF YES* → **24-hour NIHSS** (0-42 Integer)
- **Date of end of acute care** (Date)
- **Discharge Date OR Date of Death in Hospital** (Date)
- **Discharge Disposition** (Home, Rehab, LTC, Died)

### OUTCOMES
- **90-day mRS** (Date/Time) – – **90-day mRS** (0-6 Integer)
- **Return to previous function** (Yes/No)

## COMPREHENSIVE STROKE CENTRE (FROM PSC)

### PRE-TREATMENT PROCESS
- **Arrival** (Date/Time)
- **Arrival from Hospital** (From list of their spoke hospitals)
- **Was patient re-imaged at the CSC?** (Yes/No) → *IF YES* → **CT** (Date/Time)
- **Was a CTA done immediately after CT?** (Yes/No/Not done) → *IF NO* → **CTA** (Date/Time)
- **Was a CTP done immediately after CT?** (Yes/No/Not done) → *IF NO* → **CTP** (Date/Time)
- **ASPECTS Collected?** (Yes/No) → *IF YES* → **ASPECTS** (0-10 Integer)
- **Location of Occlusion** (Pick list)

### TREATMENT PROCESS
- **EVT Performed?** (Yes/No) → *IF YES* → **Arterial Access** (Date/Time); **Groin Closure** (Date/Time); **TICI Scale Final** (List); **Endovascular Procedure** (List)

*Legend:* *Primary Stroke Centre* | *Comprehensive Stroke Centre* | *Required Data Entry* | *Optional Data Entry*

OPTIMISING ACCESS

# List of Variables

## COMPREHENSIVE STROKE CENTRE *(DIRECT)*

### PATIENT INFORMATION
- **Patient Identifier**
- **Age** (Numeric)
- **Sex** (Male/Female/Other)
- **Gender** (Woman/Man/Non-binary/Other)
- **Race** (List)

### STROKE OCCURENCE INFORMATION
- **Postal Code**
- **Where did stroke occur?** (Address)
- **Last Seen Normal** (Date/Time) --- **Witnessed Onset?** (Yes/No)
- **Stroke Occurred in?** (Community/ Hospital)
- **Pre-treatment NIHSS Collected?** (Yes/No) → *IF YES* → **Pre-treatment NIHSS** (0-42 Integer)

### PRE-TREATMENT PROCESS
- **Time 911 Call** (Date/Time)
- **Arrival** (Date/Time)
- **Arrival Method** (Ambulance/Walk-in/Transfer)
- **CT** (Date/Time)
- **Was a CTA done immediately after CT?** (Yes/No/Not done) → *IF NO* → **CTA** (Date/Time)
- **Was a CTP done immediately after CT?** (Yes/No/Not done) → *IF NO* → **CTP** (Date/Time)
- **ASPECTS Collected?** (Yes/No) → *IF YES* → **ASPECTS** (0-10 Integer)
- **Location of Occlusion** (Pick list)

### TREATMENT PROCESS
- **Thrombolysis Received?** (Yes/No) → *IF YES* →
  - **Thrombolysis** (Date/Time)
  - **Thrombolysis Drug?** (tPA/TNK)
- **EVT Performed?** (Yes/No) → *IF YES* →
  - **Arterial Access** (Date/Time)
  - **Groin Closure** (Date/Time)
  - **TICI Scale Final** (List)
  - **Endovascular Procedure** (List)

### ACUTE CARE
- **NIHSS at 24 hours collected?** (Yes/No) → *IF YES* → **24-hour NIHSS** (0-42 Integer)
- **Date of end of acute care** (Date)
- **Discharge Date OR Date of Death in Hospital** (Date)
- **Discharge Disposition** (Home, Rehab, LTC, Died)

### OUTCOMES
- **90-day mRS** (Date/Time) --- **90-day mRS** (0-6 Integer)
- **Return to previous function** (Yes/No)
