# Supplementary material for: Identification of Design Requirements for a Software Application for Use by Clinicians That Collects Acute Stroke Treatment Data During Clinical Workflow: Pilot Study
Source: JMIR Form Res. 2025 Dec 19;9:e64800. doi: 10.2196/64800 (PMC12759296; doi:10.2196/64800)

Figure S1: Information Architecutre to define the layout and organization of the prototype.


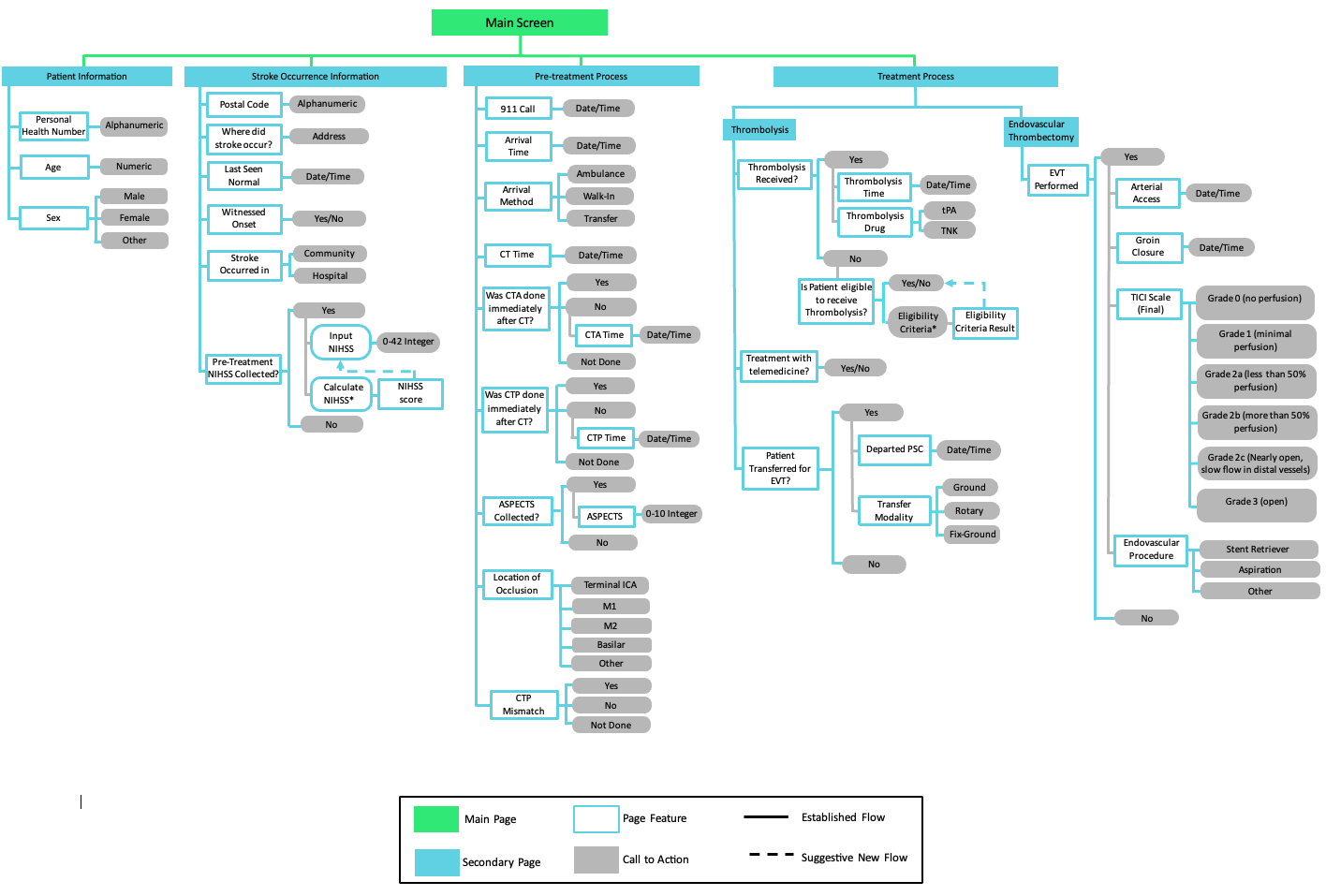


Figure S2: Sub Information Architecture for NIHSS


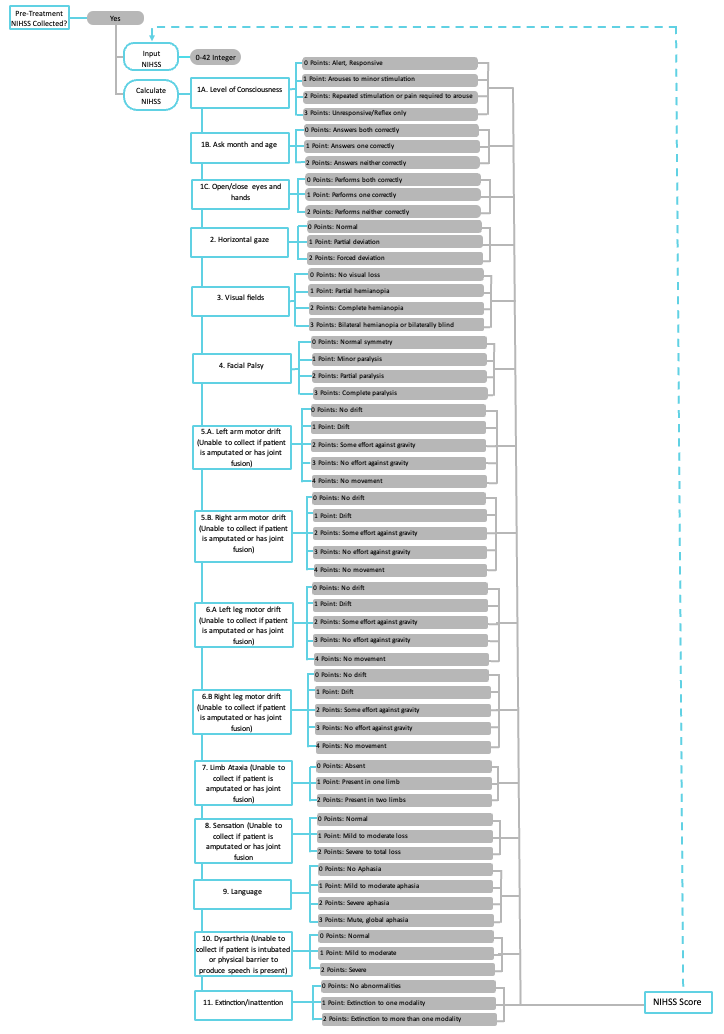


Figure S3: Sub Information Architecture for Thrombolysis Eligibility Criteria


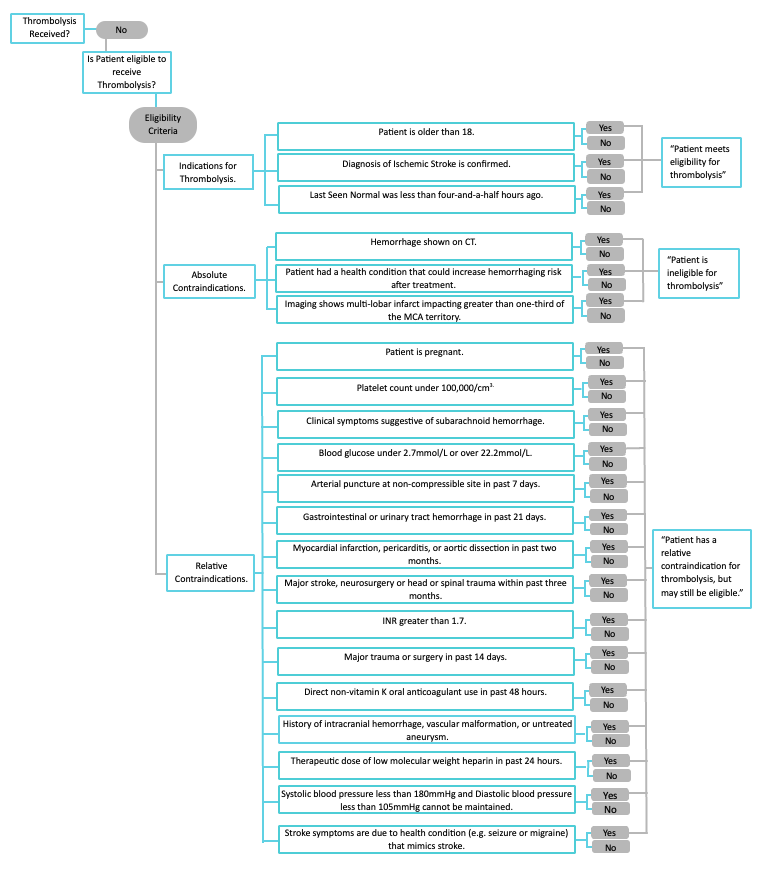

Supplement: Multimedia Appendix 2 [file formative_v9i1e64800_app2.docx]
