# Supplementary material for: Identification of Design Requirements for a Software Application for Use by Clinicians That Collects Acute Stroke Treatment Data During Clinical Workflow: Pilot Study
Source: JMIR Form Res. 2025 Dec 19;9:e64800. doi: 10.2196/64800 (PMC12759296; doi:10.2196/64800)
Supplement: Multimedia Appendix 3 [file formative_v9i1e64800_app3.pdf]

Supplemental: Semi-structured Interview guide

Part 1: General Feedback

1. As you ran through the scenario, what were your thoughts and feedback on the prototype?
2. If you could change anything about the prototype, what would you change?

Part 2: Layout and Information Organization

3. Please provide feedback on the layout, and how the information was organized?
    a. Did you feel the data or data entry fields were in the right sections?
4. After you entered the data, there was a summary page, please provide feedback on it.
    a. Additionally, would you please provide feedback on other sections of the prototype (only sections that were not previously discussed) such as:
        i. The main header
        ii. The NIHSS calculator
        iii. The sidebar summary
        iv. The different data entry field interaction styles
        v. The inclusion exclusion criteria

Part 3: Considerations Towards Integrating Software into Workflow

5. What are your thoughts about using the prototype on an iPad?
6. Please provide your thoughts on entering data on the prototype during workflow.
    a. How would you feel if you used this software during a real stroke treatment?
    b. How much do you think using this software would affect your workflow?
    c. What might help the prototype more effectively integrate into your workflow?

Part 4: Final Comments

7. What did you like most about the prototype? Why?
8. What did you like least about the prototype? Why?
9. What other changes would you make to the prototype? Why?
10. What would you keep for future prototype iterations? Why?
